# Aquatic Floristic Surveys Reveal Overlooked Alien Macrophytes in Rice Fields of Northern Italy: *Najas chinensis* (Hydrocharitaceae) and *Chara fibrosa* s.l. (Characeae)

**DOI:** 10.3390/plants15213387

**Published:** 2026-11-01

**Authors:** Dario Salemi, Rosi De Luca, Vincenzo Ilardi, Anna Geraci, Rosario Schicchi, Angelo Troia

**Affiliations:** 1 Dipartimento di Scienze della Terra e del Mare (DISTEM), Università degli Studi di Palermo, 90123 Palermo, Italy; dario.salemi@unipa.it; 2 Dipartimento di Scienze e Tecnologie Biologiche, Chimiche e Farmaceutiche (STEBICEF), Università degli Studi di Palermo, 90123 Palermo, Italy; rosi.deluca@unipa.it (R.D.L.); vincenzo.ilardi@unipa.it (V.I.); anna.geraci@unipa.it (A.G.); 3 Dipartimento Scienze Agrarie, Alimentari e Forestali (SAAF), Università degli Studi di Palermo, 90128 Palermo, Italy; rosario.schicchi@unipa.it; 4 National Biodiversity Future Center (NBFC), Piazza Marina 61, 90133 Palermo, Italy

**Keywords:** agroecosystems, botanical monitoring, charophytes, *Chara pelosiana*, *Chara benthamii*, cryptic invasions, early detection, Piedmont, Po Plain

## Abstract

The alien flora of rice fields and associated aquatic habitats remains poorly documented. Surveys conducted in 2024–2025 in eastern Piedmont (Northern Italy) recorded several alien macrophytes. Here, we focus on two taxa: *Najas chinensis* N. Z. Wang (Magnoliophyta, Hydrocharitaceae) and *Chara fibrosa* C. Agardh ex Bruzelius s.l. (Charophyta, Characeae). In Italy, *N. chinensis* had previously been known from a single herbarium specimen collected in 2004 and recognised only years later through molecular analyses. We found it at more than half of the surveyed sites, suggesting that its apparent rarity may reflect limited floristic exploration rather than a genuinely restricted distribution. We also provide an identification key to the Italian species of *Najas*. *C. fibrosa*, a tropical–subtropical species historically associated with European rice fields and only sporadically recorded in Italy since the nineteenth century, is reported here for the first time from Piedmont, confirming its occurrence in the western Po Plain. Our findings show that even limited floristic surveys can reveal overlooked alien aquatic plants and improve knowledge of their distribution, thus highlighting the need to strengthen field monitoring activities.

## 1. Introduction

Freshwater ecosystems are among the most biologically diverse and ecologically valuable environments on Earth, providing essential ecosystem services such as water purification, nutrient cycling, carbon sequestration, and habitat for a wide range of organisms [1,2]. Despite covering less than 1% of the Earth’s surface, they support approximately 10% of all known species and nearly one-third of vertebrate diversity. Simultaneously, freshwater ecosystems are experiencing some of the fastest rates of biodiversity loss globally owing to multiple anthropogenic pressures, including habitat degradation, eutrophication, hydrological alteration, pollution, biological invasions, and climate change [1,2,3].

Long-term biodiversity monitoring provides the foundation for understanding ecological change, evaluating conservation measures, and supporting evidence-based management. Nevertheless, freshwater monitoring programs have traditionally focused on faunal groups, particularly fish, macroinvertebrates, and amphibians, whereas aquatic flora has received comparatively less attention [4]. This taxonomic imbalance is striking considering the central ecological role of aquatic plants in maintaining ecosystem functioning. Aquatic macrophytes regulate nutrient cycling, stabilize sediments, influence hydrological processes, provide habitat and food resources for numerous organisms, and respond sensitively to environmental disturbances, making them valuable indicators of ecological status [5,6]. Consequently, the current geographic fragmentation and temporal discontinuity of floristic surveys leave critical gaps in our understanding of baseline species distributions and community dynamics.

Addressing these knowledge gaps increasingly relies on advanced tools such as remote sensing, ecological modeling, and biodiversity databases. However, such technologies cannot fully replace ground-truth field observations, which remain indispensable for detecting overlooked taxa and validating distribution models. Paradoxically, despite this critical need, field-based ecological research has declined markedly in recent decades. This shift is driven in part by institutional and publication systems that favor computational and synthetic studies over empirical field monitoring [7,8].

The need for systematic monitoring of freshwater flora has become even more urgent in the context of biological invasions. Aquatic ecosystems are particularly vulnerable to the establishment and spread of invasive alien plant species, which can rapidly alter community composition, ecosystem functioning, water quality, and habitat structure, while generating substantial ecological and economic impacts [9,10,11]. Early detection of invasive aquatic plants is widely recognized as one of the most effective strategies for limiting their spread and reducing management costs, yet this requires regular, standardized, and taxonomically comprehensive floristic surveys [12].

In this context, this study presents the results of targeted surveys of aquatic flora in rice paddies in eastern Piedmont (Northern Italy). Conducted within the framework of a short-term project, this work aims to contribute to the knowledge of freshwater plant diversity and highlights the importance of integrating comprehensive floristic monitoring into routine freshwater biodiversity assessment programs.

Notably, while several non-native taxa were recorded, the surveys led to the detection of two alien species of particular interest: the vascular plant *Najas chinensis* N. Z. Wang (=*Najas orientalis* Triest & Uotila ≡ *Caulinia orientalis* (Triest & Uotila) Tzvelev), hitherto known in Italy on the basis of a single specimen [13], and the charophyte alga *Chara fibrosa* C. Agardh ex Bruzelius s.l. (=*Chara fibrosa* subsp. *benthamii* (A. Braun) Zaneveld ≡ *Chara benthamii* A. Braun = *Chara baueri* f. *pelosiana* (Avetta) R. D. Wood ≡ *Chara pelosiana* Avetta) previously unrecorded in the Piedmont region and supposed to be rare throughout the Po Valley [14].

## 2. Material and Methods

The specimens were collected during two sampling campaigns conducted from 9 to 11 September 2024 and 16 to 18 June 2025 in rice paddies and adjacent wetlands located in the provinces of Novara and Vercelli, eastern Piedmont (Italy). This study was carried out within the framework of the interdisciplinary project Innovarisi (Italian National Recovery and Resilience Plan, PNRR M4C2 Inv.1.4, CUP D13C22001330005) [15].

Sampling was performed across nine selected stations located within limited areas of the Vercelli and Novara provinces, with one site extending slightly into the adjacent Biella province (Figure 1), and was designed to compare conventionally cultivated rice paddies, organic rice paddies and natural wetlands [15]. The study sites lie in the western sector of the Po Plain, north of the Po River, within a broad irrigated agricultural landscape that includes nearly the entire province of Vercelli, the central-southern portion of Novara, the central-northern sector of Pavia, and, to a lesser extent, parts of Alessandria, Biella, and Milan provinces. This region represents one of the largest irrigated agricultural landscapes in Europe and is characterised by a complex mosaic of rice paddies, canals, irrigation ditches, sluices, marshes and riverine water bodies (including the Dora Baltea, Sesia, and Ticino; as well as the Agogna, Cervo, Elvo, and Terdoppio streams) that sustain the hydrological system. The irrigation network exploits a gentle natural gradient from west to east, with the main distribution channels (Cavour Canal, Naviglio d’Ivrea, and Depretis Canal) conveying water from April to June to flood the rice fields.

The collected specimens were dried and deposited in the collectors’ private collection and in the Herbarium Mediterraneum Panormitanum in Palermo (PAL) (herbaria codes according to Thiers [16]). Taxonomic nomenclature for vascular plants follows the World Checklist of Vascular Plants [17]. The nomenclature and species concepts applied to charophytes follow the treatment in *Charophytes of Europe* [18]. *Najas* specimens were identified using systematic monographs [19,20] and specific literature [21,22,23]. Furthermore, the recent literature on the taxonomy and systematics of the genus was reviewed in order to propose a simplified key for the identification of the species present in Italy.

The geographical coordinates used in this work follow the World Geodetic System standard (WGS84) and are expressed in decimal degrees with a precision of 5 decimal places. The map was created using QGIS 3.40 software.

## 3. Results

### 3.1. Area of Study

Within the investigated area, *Najas chinensis* was recorded in five of the nine monitored stations, while *Chara fibrosa* s.l. was found in three. The two species co-occurred at two sites.

The stations where both species were recorded cluster in two main areas: (A) sites adjacent to the “Riserva naturale della Palude di Casalbeltrame” (municipalities of Biandrate and Casalino, Novara province) and (B) sites surrounding the “Riserva naturale Palude di San Genuario” (municipalities of Crescentino and Fontanetto Po, Vercelli province). Conversely, in macro-area C, located close to the municipality of Rovasenda (across the Vercelli and Biella provinces), neither of the two species was detected.

During the September 2024 campaign, *N. chinensis* was recorded at A1, A3, B1, and B2 sites, while *C. fibrosa* was recorded at A1, B2, and B3. A subsequent survey in June 2025 did not confirm the previously recorded occurrence but revealed a new population of *N. chinensis* at B3. This failure to re-detect previously recorded populations is related to the highly dynamic nature of rice paddy plant communities, which change rapidly in response to temporal variation in agronomic practices (e.g., soil preparation, pesticide application, weed control, alternating flooding and drying cycles), which naturally favour highly specialized taxa [24,25,26,27].

Both species occurred in cultivated rice paddies as well as in adjacent semi-natural habitats. In detail, the semi-natural site is the “Riserva naturale Palude di San Genuario” (included in the Natura 2000 network with the code IT1120007): it was used as a fish farm until the early 1990s and, following the cessation of production activities, has progressively naturalized [28]. Details on site locations and environmental characteristics are provided in Table 1.

### 3.2. Morphological Identification

Specimens of *Najas* collected in this study are monoecious, with stems and the abaxial surface of the leaves lacking spines. The leaf sheaths are rounded, and septa are present. Male flowers are enclosed in a spathe, whereas female flowers are naked. Anthers are four-locular. The areoles are regularly arranged in longitudinal rows of 20–30, with a squarish shape and raised cell walls. These morphological features allowed for their correct identification as *Najas chinensis* (Figure 2).

Specimens of aplostephanous monoecious *Chara* collected in this study show a diplostichous, isostichous to tylacanthous cortication, and naked branchlets terminating in an end-cell surrounded by a group of bract-cells (Figure 3). These features allowed for their identification as *Chara fibrosa* s.l. according to Schubert et al. [18]. The specimens exhibit 1–2 stipulodes per branchlet, a character shared by *C. pelosiana* sensu Langangen [29] and *C. benthamii* Braun & Nordstedt (1882). Within *C. fibrosa* s.l., the observed combination of characters shows a greater affinity with the morphotype referred to as *C. benthamii*, particularly in the length of the stipulodes, which ranges from one-half to one-third of the length of the basal branchlet cell, and in the black colour of the oospores. However, the oospore dimensions observed in our specimens (approximately 300 µm long × 200 µm wide) are considerably smaller than those reported for *C. benthamii* and are more similar to those reported for the morphotype referred to as *C. pelosiana*. The latter is characterized by brown oospores according to Langangen [29], although the author noted uncertainty regarding their maturity (observed in the type specimen). Gametangia were observed up to the fourth branchlet nodes. The occurrence of gametangia at the fourth node extends beyond the third-node position reported in the literature, although this difference should be interpreted with caution because this character was observed in specimens cultivated under laboratory conditions.

Deposited specimens:

*Chara fibrosa* s.l.: Italy, Vercelli, pond in the nature reserve “Riserva naturale Palude di San Genuario”, (45.2192, 8.17853), (10 September 2024), leg. A. Troia, det. D. Salemi, R. De Luca, A. Troia (PAL).

*Najas chinensis*: Italy, Novara, rice field near the nature reserve “Palude di Casalbeltrame”, (45.4411, 8.49625), (9 September 2024), leg. A. Troia, det. D. Salemi, R. De Luca, A Troia (PAL); Italy, Vercelli, pond in the nature reserve “Riserva naturale Palude di San Genuario”, (45.2192, 8.17853), (10 September 2024), leg. A. Troia, det. D. Salemi, R. De Luca, A. Troia (PAL).

Floristic records of aquatic macrophytes from stations where the species occurs are presented in Table 2.

## 4. Discussion

### 4.1. Taxonomic Remarks and Current Status of Najas chinensis in Italy

*Najas* L. is a cosmopolitan genus of the Hydrocharitaceae family, comprising about 40 species of submerged aquatic macrophytes distributed from temperate to tropical regions [23]. Most species occur in tropical Asia, which has been previously proposed as the area of origin of the genus [13]. However, recent phylogenetic analyses suggest North America as the ancestral region instead [13].

All species of *Najas* are characterized by fragile filiform stems, linear one-veined leaves, and unbranched adventitious roots [23]. Because of their extremely reduced reproductive structure, the last monograph of the Old World species [20] relied primarily on the leaf teeth and sheath morphology, the presence or absence of septa or fibers in the leaf blade, the arrangement and pattern of seed coat pitting, and the number of anther locules for species identification. The genus *Najas* in Italy includes several taxa belonging to both subgenera: subgenus *Najas* (*N. major* All., *N. marina* L. subsp. *marina,* and *N. marina* subsp. *armata* Horn) and subgenus *Caulinia* Willd. (*N. chinensis* N. Z. Wang, *N. gracillima* (Engelm.) Magnus, *N. graminea* Delile, and *N. minor* All.). According to Hussner [30], who reviewed alien aquatic species in Europe, all *Caulinia* species are considered alien except *N. minor*.

*Najas chinensis* is an aquatic plant native to freshwater habitats across south-central and southeastern China, Hainan, Japan, Korea, Manchuria, Primorye and Taiwan [31]. In its native range *N. chinensis* is primarily associated with rice paddies [32], although non-typical occurrences have been reported (e.g., a large roadside ditch in Turkey [21]).

Outside eastern Asia, *Najas chinensis* has been reported from several countries in Europe and Asia (Table 3). The first report outside its native range was provided by Triest & Uotila [21], who described it as a new species (*N. orientalis*) in European Turkey, based on a specimen collected in 1972. Later, Ito et al. [13] clarified that the species had already been described as *N. chinensis* [33], which therefore has taxonomic priority. Subsequent records include Greece [34] and the northern Caucasus [35], still published under the name *N. orientalis*.

In Italy, the species was first reported in 2017: during a phylogenetic revision of the genus, Ito et al. [13] discovered that a specimen collected in 2004 within rice fields in the province of Pavia and previously identified as *N. gracillima* was genetically attributable to *N. chinensis*. Since then, the species has been included in the Italian flora [36], although this record relies on a single specimen and lacks ecological or distributional information. Ardenghi [37], in his recent work on the flora of Pavia province, lists *N. chinensis* as a very rare (RR) species living in the *Oryzo sativae-Echinochloion oryzoidis* O. de Bolòs et Masclans 1955 community. Notably, the Italian occurrence is not yet included in the POWO database.

Our detection of *N. chinensis* in more than half of the surveyed stations points to a much wider local distribution than previously assumed. The vast, contiguous area of Lombard–Piedmont rice cultivation provides an abundance of suitable habitats, likely facilitating secondary spread through multiple dispersal vectors. The dense network of irrigation canals and ditches promotes passive downstream hydrochory along the west–east hydrological gradient. Concurrently, waterbirds may enable upstream or overland zoochory, while contaminated rice seed batches used for sowing represent a highly plausible pathway for both the primary anthropogenic introduction and subsequent agricultural dispersal of alien *Najas* species in Italy.

To facilitate further monitoring, we present here a dichotomous key for the Italian *Najas* species, alongside differential characters distinguishing *N. chinensis* from other members of the subgenus *Caulinia*.

Key for the identification of taxa of the genus *Najas* occurring in Italy

(1) Plants dioecious; stems and abaxial surface of leaves usually spiny; areoles on seeds irregularly arranged ………………………………………………… 2 *Najas* subg. *Najas*

(1) Plants monoecious; stems and abaxial surface of leaves usually not spiny; areoles on seeds longitudinally arranged ………………………………… 4 *Najas* subg. *Caulinia*

(2) Seeds (3.3–)4–6(–7.5) mm long; ovary (3–)4–6 × 2–2.5 mm; style and stigma 2–3 mm long; anther 3.8–4 mm long …………………………………………………… *Najas major*

(2) Seeds 2.5–4(–5.1) mm long; ovary 0.7–3.4 × 0.4– 1.7 mm; style and stigma 0.2–1.4 mm long; anther 1–3.3 mm long ………………………………………………………… 3

(3) Stems and back of midrib not very spiny, (0–)2–10 (–15) spines in 2 cm; seeds 3.7–4.3 mm long …………………………………………………… *Najas marina* subsp. *marina*

(3) Stems and back of midrib very spiny, (10–)15–30 (–50) spines in 2 cm; seeds 2.5–3.5 mm long …………………………………………………… *Najas marina* subsp. *armata*

(4) Anthers 1-locular; sheath slightly auriculate or truncate ……………………… (5)

(4) Anthers 4(or rarely 2)-locular; sheath deeply auriculate or rounded ………… (6)

(5) Seeds with many transversely elongated ladderlike pits; areoles broader than long, fusiform to hexagonal; fruit narrowed and slightly curved at apex ………. *Najas minor*

(5) Seeds with many longitudinally elongated pits; areoles longer than broad, rectangular or polygonal; fruit usually straight …………………………………. *Najas gracillima*

(6) Sheath deeply auriculate; leaves without septa and with fibres; male and female flowers without a spathe; cell wall of the fruits not raised; areoles squarish to hexagonal or rectangular, 25–35 (60) per row…………………………………………………………………………………. *Najas graminea*

(6) Sheat rounded; leaves with septa and without fibres; male flowers only in a spathe; cell wall of the fruits raised; areoles squarish, 20–30 per row ……………………………………………………………………………………… *Najas chinensis*

### 4.2. Chara fibrosa: Taxonomic and Distributional History in Italy

*Chara fibrosa* belongs to *Chara* sect. *Agardhia*, a taxonomically challenging lineage that has historically included more than twenty described species, subspecies and infraspecific taxa, distributed predominantly in tropical and subtropical regions, particularly throughout Asia and Australia [38]. Species assigned to this section share a distinctive combination of characters, including corticated axes, ecorticate branchlets, a single row of stipulodes, clusters of bract cells subtending the terminal branchlet segment, and the absence of gyrogonites [38]. By contrast, European representatives of the section are extremely rare and geographically isolated, having been recorded only from a few localities, mostly associated with rice fields or other temporary wetlands [38,39].

The taxonomy of section *Agardhia* has undergone repeated revisions over the last few decades and remains far from being resolved. The broad concept of *Chara fibrosa* adopted by Wood & Imahori [40], which merged numerous previously described species into a single, morphologically variable taxon, is now regarded as an oversimplification of the diversity within the group. Recent morphological and nomenclatural studies instead support a narrower interpretation of *C. fibrosa* sensu stricto while recognising that a comprehensive revision of section *Agardhia*, integrating morphological and molecular evidence, is still required before the relationships among many taxa can be confidently resolved [38].

The taxonomic interpretation of the European representatives of this complex has changed substantially through time. Plants collected in rice fields near Modena in 1886 were originally described by Avetta in 1898 as the distinct species *Chara pelosiana*. This interpretation remained largely overlooked until Langangen [29] re-examined historical material and regarded *C. pelosiana* as conspecific with *C. fibrosa*, a treatment subsequently followed by several authors. Thus, plants discovered in rice fields near Ferrara in 1999 and later populations reported from southern France were identified as *C. fibrosa* subsp. *benthamii* according to the classification proposed by Zaneveld [39,41,42].

Casanova [38] and Soulié-Märsche & Abdelahad [39] concluded that the European representatives of section *Agardhia* can be referred to *Chara fibrosa* s.l. including at least two distinct morphological entities: the historical Italian morphotype described as *C. pelosiana* and a second morphotype formerly identified as *C. fibrosa* subsp. *benthamii*. Neither of these can presently be assigned with confidence to *C. fibrosa* sensu stricto, highlighting the need for a comprehensive taxonomic revision of the entire section.

Against this taxonomic background, the specimens examined in this study represent a new occurrence of *Chara fibrosa* s.l. in Northern Italy. Their combination of morphological characters, 1–2 stipulodes per branchlet, and particularly the stipulodes length and the black colour of the oospores, shows a greater affinity with the morphotype formerly referred to as *C. benthamii*. The relatively small oospores observed in our specimens, however, do not fully correspond to the dimensions reported for the *C. benthamii* morphotype, highlighting the morphological variability and taxonomic uncertainty within the *C. fibrosa* complex. The new record represents the first documented occurrence of this lineage in Piedmont and extends its known distribution within the western Po Plain.

### 4.3. Implications for Field Monitoring and Conservation Assessment

The findings of this study highlight the complementary roles of field surveys and taxonomic research in documenting aquatic biodiversity and monitoring biological invasions.

Repeated floristic investigations are essential not only to detect previously unrecorded species, but also to track changes in their distribution and distinguish actual range expansion from patterns resulting from incomplete sampling or delayed identification. Indeed, the interval between the introduction of an alien species and its first documented occurrence may be difficult to establish when historical records remain unidentified or are published long after collection, potentially biasing the reconstruction of invasion histories and estimates of invasion lag [43,44,45].

Accurate taxonomic identification is equally important because floristic records may directly inform conservation assessments and management decisions. In the San Genuario Reserve, for example, the previous record of a *Chara* sp. contributed to the inclusion of EU habitat 3140, “Hard oligo-mesotrophic waters with benthic vegetation of *Chara* spp.”, in the reserve’s management plan [28]. If the corresponding *Chara* population is subsequently shown to represent an alien taxon, this raises an important management question: to what extent can the occurrence of an alien species influence the interpretation, conservation status, and management priorities of habitats defined partly on the basis of their vegetation?

Rice fields and their associated irrigation networks represent particularly relevant environments for such investigations. These highly dynamic agroecosystems may provide suitable habitats for otherwise overlooked aquatic plants while also facilitating the introduction and dispersal of alien taxa [46,47]. Their hydrological connectivity, together with seasonal flooding, drainage and agricultural management practices, can influence both the occurrence and detectability of aquatic macrophytes. Monitoring should therefore extend beyond cultivated fields to include irrigation channels, ditches and adjacent semi-natural wetlands, ideally through surveys conducted at different times and, where possible, repeated across years. Such an approach would improve the detection of inconspicuous or intermittently occurring species and help clarify their distribution and persistence within agricultural landscapes.

## 5. Conclusions

The survey of only nine sampling stations in eastern Piedmont revealed two alien aquatic macrophytes of biogeographical and taxonomic interest. *Najas chinensis*, previously known in Italy from a single specimen collected in 2004 and identified as such only after molecular analyses, was recorded at five stations, suggesting that its occurrence in the region has been substantially under-recorded. *Chara fibrosa* s.l. is reported here for the first time from Piedmont. Although its morphological characters show a greater affinity with the morphotype formerly referred to as *C. fibrosa* subsp. *benthamii*, discrepancies with published descriptions prevent a definitive taxonomic assignment, provide further evidence of the morphological complexity of the *C. fibrosa* complex and reinforce the need for a comprehensive revision of section *Agardhia*.

These findings demonstrate that targeted field surveys can reveal overlooked species while providing evidence relevant to taxonomic research and conservation assessment. Regular field-based monitoring, combined with rigorous taxonomic verification, is therefore essential to improve knowledge of aquatic plant distributions, detect alien taxa, and support informed conservation and management of freshwater ecosystems.

## Figures and Tables

**Figure 1:**
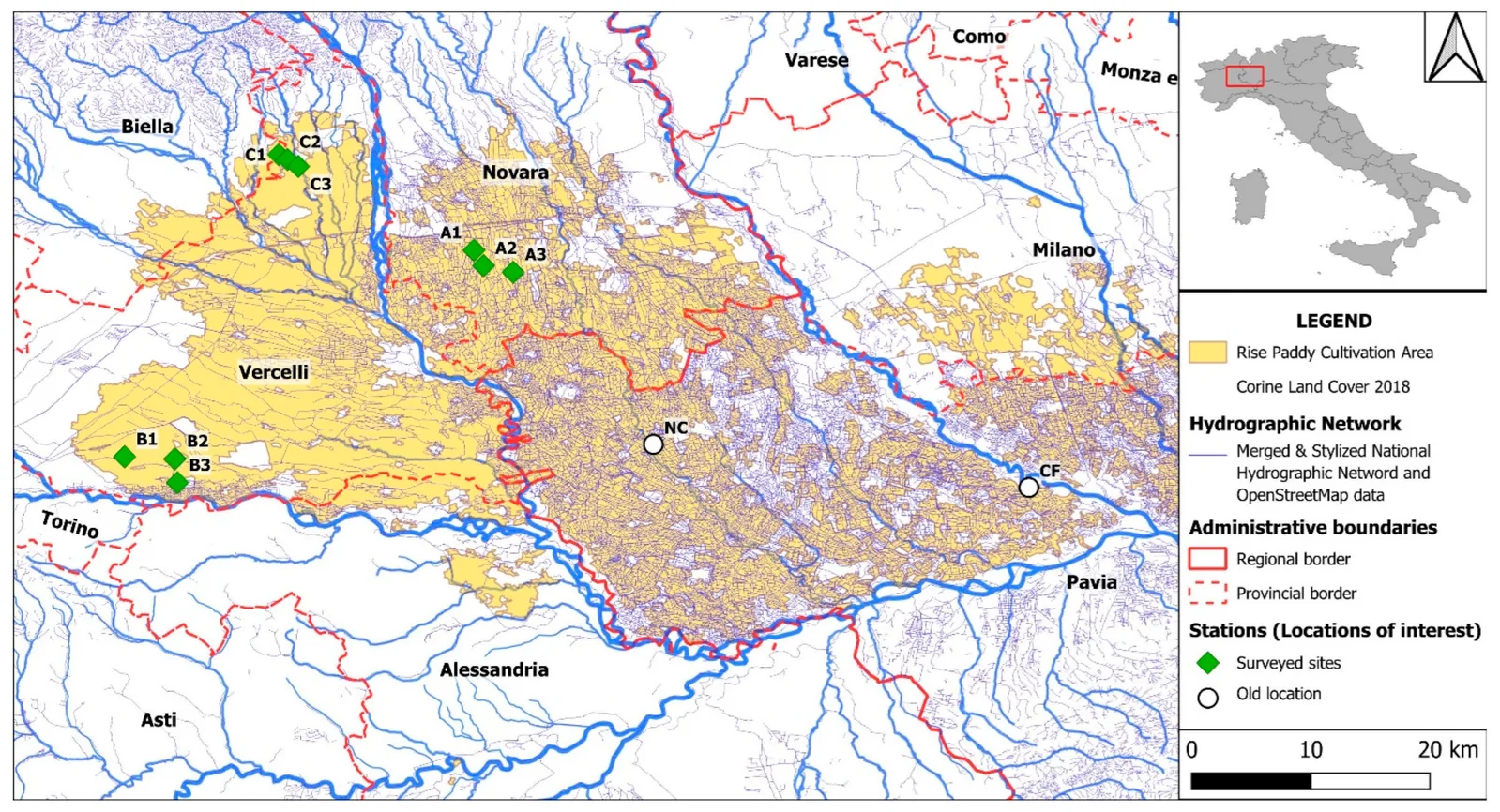
Overview of the study area showing rice cultivation and hydrographic networks. The yellow polygon indicates rice paddy areas based on the *Corine Land Cover 2018* dataset. The blue hydrographic network was obtained by merging and stylizing the national hydrographic network with OpenStreetMap data, highlighting secondary channels and irrigation ditches (*rogge*). Administrative boundaries are marked by solid red lines (regional borders) and dashed red lines (provincial borders). Green diamonds represent surveyed sites, while white circles indicate previously known stations (CF = *Chara fibrosa*; NC = *Najas chinensis*). Letters indicate the main sampling areas: A = Riserva Naturale della Palude di Casalbeltrame; B = Riserva Naturale Palude di San Genuario; C = Rovasenda. For each area, three different environments (a conventional rice field, an organic rice field and a semi-natural pond) were sampled. Note: Provincial labels (e.g., Biella, Novara, etc.) are centered on the respective visible portions of the polygons and do not correspond to exact city locations.

**Figure 2:**
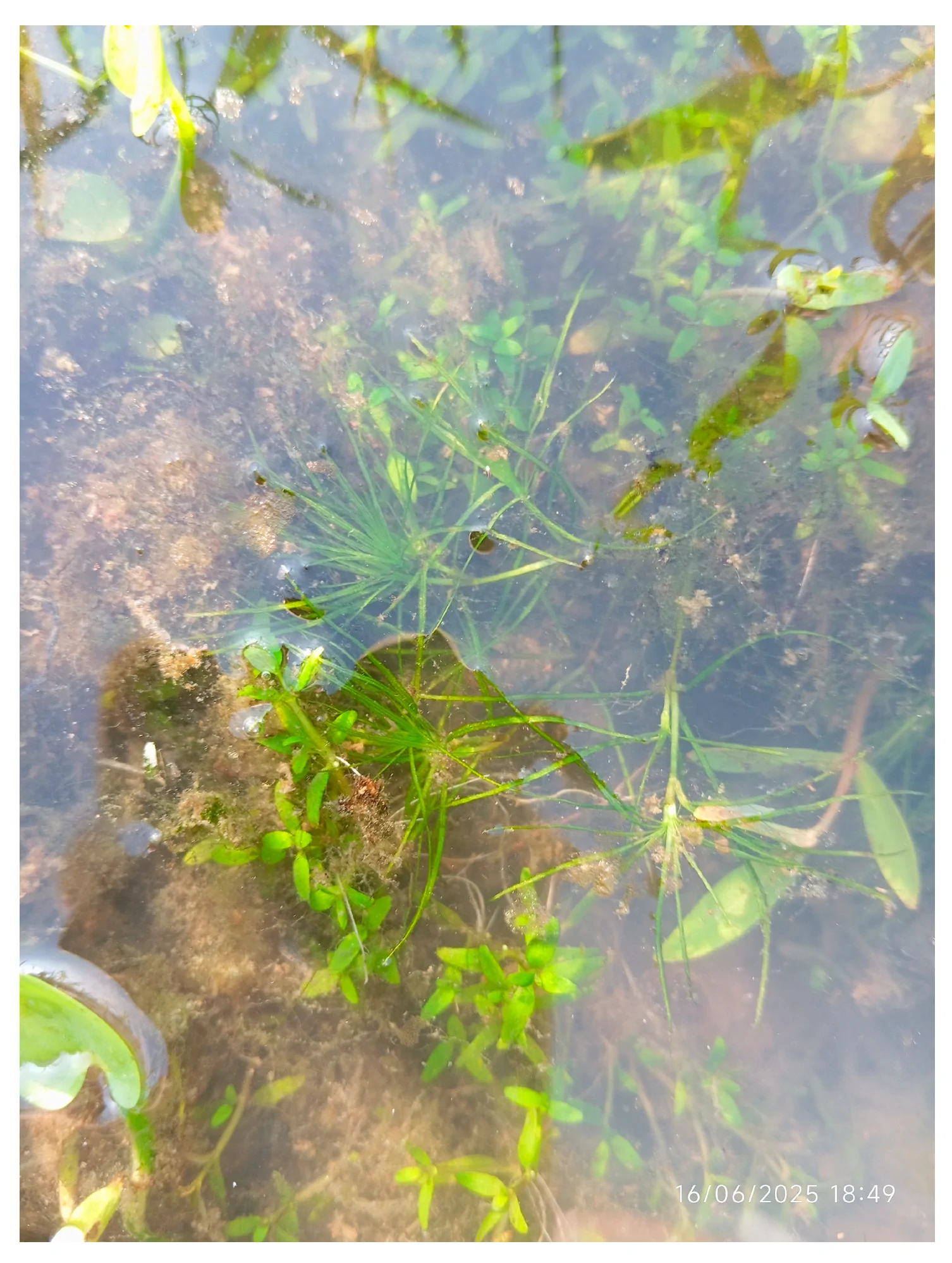
Habit of *Najas chinensis* specimens in a rice paddy of the study area.

**Figure 3:**
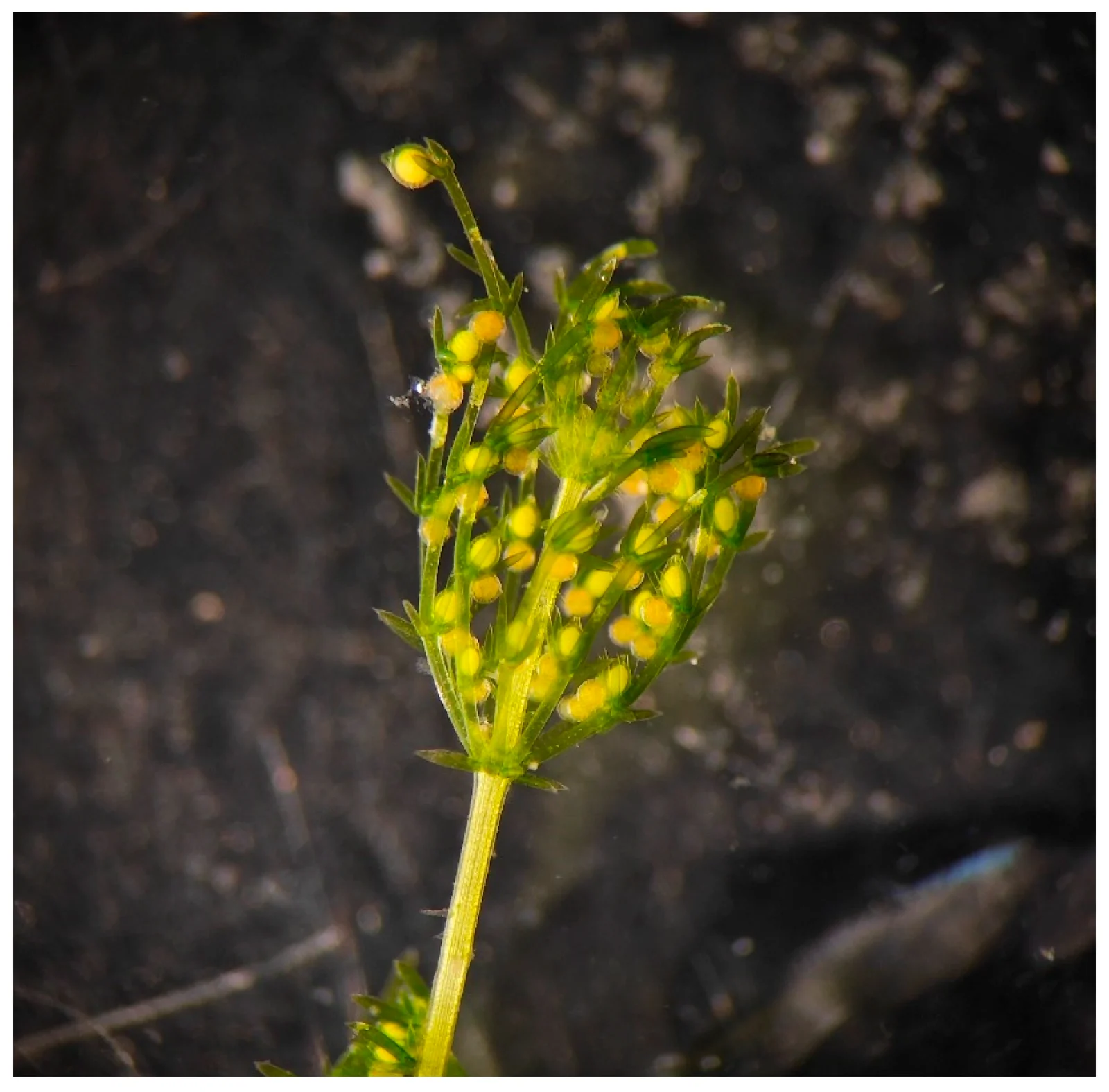
Morphological detail of the upper whorl and branchlet arrangement in one individual of *Chara fibrosa* s.l. from the study area.

**Table 1:** Geographic locations, site types, and environmental parameters (dissolved oxygen, water temperature, pH, electrical conductivity, and salinity) of the sampled stations.

Province	Macro-Area	Site Code	Site Type	Latitude	Longitude	Date	DO (mg/L)	Water T (°C)	pH	EC (μS)
Novara	A Riserva naturale della Palude di Casalbeltrame	A1	Conventional rice field	45.44112	8.49625	9 September 2024	4.02	22.3	7.3	370
		A3	Organic rice field	45.41782	8.53798	9 September 2024	1.44	32.1	7.45	263
Vercelli	B Riserva naturale Palude di San Genuario	B1	Organic rice field	45.22147	8.12485	10 September 2024	1.7	25.5	7.02	321
		B2	Semi-natural pond	45.21921	8.17853	10 September 2024	4.5	20.3	7.5	312
		B3	Conventional rice field	45.19395	8.18112	16 June 2025		27.6	8.3	34.88
						10 September 2024	3.85	22.4	7.1	397

**Table 2:** Floristic records of aquatic macrophytes from sampled stations with occurrences of *Najas chinensis* and *Chara fibrosa*.

Site	A1	A3	B1	B2	B3	B3
	9 September 2024	9 September 2024	10 September 2024	10 September 2024	10 September 2024	16 June 2025
*Najas chinensis* N. Z. Wang	**+**	**+**	**+**	**+**		**+**
*Chara fibrosa C*. Agardh ex Bruzelius s.l.	**+**			**+**	**+**	
*Alisma plantago-aquatica* L.					**+**	**+**
*Ammannia coccinea* Rottb.		**+**			**+**	
*Butomus umbellatus* L.		**+**				
*Cyperus difformis* L.					**+**	
*Echinochloa crus-galli* (L.) P. Beauv.			**+**		**+**	
*Heteranthera reniformis* Ruiz & Pav.	**+**	**+**	**+**		**+**	**+**
*Lemna minor* L.	**+**	**+**		**+**	**+**	**+**
*Marsilea quadrifolia* L.			**+**		**+**	
*Najas gracillima* (A. Braun ex Engelm.) Magnus			**+**			
*Ottelia alismoides* (L.) Pers.			**+**			
*Persicaria lapathifolia* (L.) Delarbre			**+**			
*Potamogeton natans* L.				**+**		
*Schoenoplectiella mucronata* (L.) J. Jung & H. K. Choi			**+**			
*Spirodela polyrhiza* (L.) Schleid.				**+**	**+**	**+**
*Chara braunii* C. C. Gmelin						**+**
*Chara* sp.		**+**	**+**	**+**		
*Nitella syncarpa* (Thuill.) Chev	**+**	**+**				

**Table 3:** Chronology of the first collection and publication records of *Najas chinensis* outside its native range, including the publication delay (Δt) in years and the respective literature sources.

	First Collection	First Published Report	Δt	Source
European Turkey	1972	1986	14 y	[21]
Greece, Peloponnesus	2001	2002	1 y	[34]
Caucasus	1970	2006	36 y	[35]
Italy, Lombardy	2004	2017	13 y	[13]

## Data Availability

The original contributions presented in this study are included in the article. Further inquiries can be directed to the corresponding author.
